# Decisive diagnostic clue for infectious abdominal aortic aneurysm caused by *Arthrobacter russicus* in a diabetic elderly woman with renal dysfunction: A case report and literature review

**DOI:** 10.3389/fcvm.2022.1007213

**Published:** 2022-10-28

**Authors:** Hiroyuki Yamamoto, Yasuto Fukushima, Yoshihiko Ikeda, Tomoyuki Suda, Mieko Goto, Jun Isogai, Toru Hashimoto, Takashi Takahashi, Hidemitsu Ogino

**Affiliations:** ^1^Department of Cardiovascular Medicine, Narita-Tomisato Tokushukai Hospital, Chiba, Japan; ^2^Laboratory of Infectious Diseases, Graduate School of Infection Control Sciences and Omura Satoshi Memorial Institute, Kitasato University, Tokyo, Japan; ^3^Department of Pathology, National Cerebral and Cardiovascular Center, Suita, Japan; ^4^Department of Surgery, Narita-Tomisato Tokushukai Hospital, Chiba, Japan; ^5^Department of General Surgery, Shonan Kamakura General Hospital, Kanagawa, Japan; ^6^Department of Radiology, Asahi General Hospital, Asahi, Japan

**Keywords:** infectious aortic aneurysm, abdominal aorta, MRA, renal dysfunction, 16S rRNA, *Arthrobacter russicus*

## Abstract

Infectious aortic aneurysm (IAA) can be a rare but potentially fatal sequela of infectious inflammatory disease of the aortic wall with a high incidence of rupture. The definitive diagnosis is based on vascular imaging of the aneurysm using contrast-enhanced computed tomography (CE-CT) and identification of the causative microorganism from positive blood cultures (BCs). However, IAA remains extremely difficult to diagnose and treat in patients with prior antimicrobial treatment or with renal dysfunction. Here we describe a case of an 85-year-old woman with IAA caused by *Arthrobacter russicus* presenting with abdominal pain and fever that was initially diagnosed as a presumptive urinary tract infection and treated with empiric antimicrobial therapy. However, persistent abdominal pain with increased serological inflammation necessitated further evaluation. Unenhanced multimodality imaging considering the renal dysfunction revealed infectious aortitis of the infrarenal abdominal aorta, together with the initial culture results, leading to the tentative diagnosis of *Klebsiella pneumoniae* aortitis. Thereafter, serial monitoring with unenhanced magnetic resonance angiography (MRA) using thin-slab maximum intensity projection (TS-MIP) revealed acute aortic expansion strongly suggestive of a pseudoaneurysm that was successfully treated with early surgical repair under adequate infection control. Despite negative Gram staining and tissue culture results for the excised aortic wall, a definitive diagnosis of IAA secondary to *A. russicus* rather than *K. pneumoniae* was finally made by confirming the histologic findings consistent with IAA and the identification of *A. russicus* 16S rRNA on the resected aortic wall. The patient also developed a vascular graft infection during the postoperative course that required long-term systemic antimicrobial therapy. This case highlights the value of unenhanced MRA in the early detection of IAA in patients with renal dysfunction and the importance of a molecular diagnosis for identifying the causative microorganism in cases of culture- or tissue-negative IAA.

## Introduction

Infectious aortic aneurysm (IAA) is a rare but serious infectious inflammatory disease of the aortic wall that often requires prompt surgical intervention because of a high associated mortality rate with antimicrobial therapy alone (1). However, various obstacles, including negative blood and tissue cultures, non-specific symptoms and signs, IAA mimics, and high perioperative mortality rates of open surgery, pose a diagnostic and therapeutic challenge in patients with IAA (1–3).

## Case description

An 85-year-old woman was admitted to our hospital with a 3-day history of abdominal pain and fever. Her medical history included a cerebral infarction, Parkinson’s disease, dementia, and diabetes. Her vital signs were as follows: blood pressure, 186/86 mmHg; heart rate, 90 beats/min; blood temperature, 38.4°C; respiratory rate, 18 breaths/min; oxygen saturation, 99% on ambient air; height, 150 cm; and weight, 43.5 kg. An abdominal examination revealed no significant pulsatile mass, rebound tenderness, audible vascular murmur, or costovertebral angle tenderness except for mild tenderness in the periumbilical region. Chest X-ray findings were unremarkable. An initial laboratory workup revealed an elevated leukocyte count of 10,200/μL (neutrophils, 89.5%), elevated levels of C-reactive protein (18.04 mg/dL; normal, < 0.14 mg/dL), fasting blood glucose (188 mg/dL; normal, < 110 mg/dL), and glycated hemoglobin (8.3%; normal, < 6.0%). Severe renal dysfunction with a calculated creatinine clearance of 19.6 mL/min was observed. A urine dipstick test revealed glucose 4 +, blood 1 +, protein 1 +, and bacteria 2 +. We initially suspected gastroenteritis, pyelonephritis, or a liver abscess.

**FIGURE 1:**
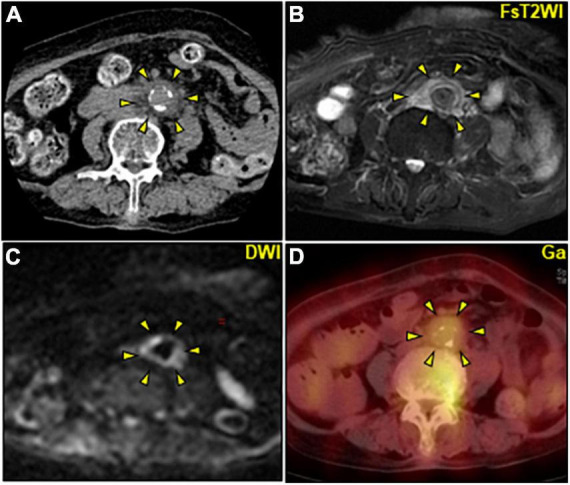
Multimodality imaging of periaortic mass of the abdominal aorta. **(A–D)** Axial images. **(A)** Unenhanced CT image showing a non-dilated abdominal aorta with a maximum diameter of 17 mm including mural calcification surrounded by circumferential heterogeneous soft-tissue density (arrowheads). **(B,C)** Unenhanced abdominal magnetic resonance image. **(B)** FsT2WI showing significantly high signal intensity and marked thickening of the periaortic tissue of the abdominal aorta (arrowheads) with inhomogeneous diffusion restriction on DWI **(C)**. **(D)** Ga-single photon emission computed tomography/CT image revealing an active accumulation of Ga (arrowheads) corresponding to the periaortic mass. CT, computed tomography; DWI, diffusion-weighted imaging; FsT2WI, fat-suppressed T2-weighted image; Ga, gallium citrate-67.

**FIGURE 2:**
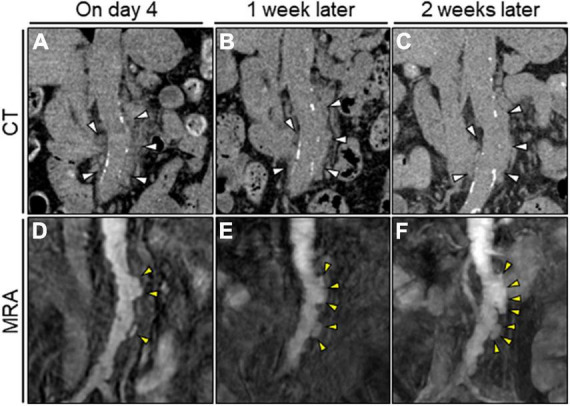
Serial unenhanced CT and MRA assessment of the abdominal aorta. **(A–C)** Serial unenhanced reconstructed coronal CT image showing no significant change in the periaortic mass of the abdominal aorta (white arrowheads). **(D)** Initial TS-MIP image of the original MRA image showing several asymmetric aortic dilatations in the abdominal aorta (yellow arrowheads). **(D–F)** Serial TS-MIP of the original MRA image showing rapid aortic expansion with multilobular protrusion of the abdominal aorta to the left side (yellow arrowheads). CT, computed tomography; MRA, magnetic resonance angiography; TS-MIP, thin-slab maximum intensity projection.

Contrast-enhanced computed tomography (CE-CT) was omitted due to the patient’s severe renal dysfunction. Abdominal ultrasonography revealed multiple gallstones and obvious intestinal gas but no intrahepatic mass, hydronephrosis, or flap in the abdominal aorta. After the collection of a urine culture and two sets of blood cultures (BCs), the patient was intravenously administered ceftriaxone 1 g daily for a presumptive urinary tract infection. Persistent abdominal pain with increased serological inflammation prompted further abdominal evaluation on day 4. Unenhanced CT revealed a non-aneurysmal dilation of the infrarenal abdominal aorta with increased soft tissue mass around the aorta (Figures 1A, 2A). Unenhanced magnetic resonance imaging (MRI) further characterized the periaortic tissue’s properties. T2 and diffusion-weighted imaging (DWI) showed a high-intensity area around the aorta with diffusion restriction (Figures 1B,C). Furthermore, gallium-67 single-photon emission computed tomography (Ga-SPECT)-CT showed abnormal uptake in the corresponding area, confirming active inflammation (Figure 1D).

We initially suspected inflammatory aortitis, including autoimmune- or immunoglobulin G4-related disease (IgG4-RD). However, serum immunological examination results were within normal ranges, suggesting less likelihood of inflammatory diseases. Urine culture and BCs conducted on admission revealed the presence of *Klebsiella pneumoniae* susceptible to conventional antimicrobials but naturally resistant to ampicillin. Accordingly, a tentative diagnosis of *K. pneumoniae* abdominal aortitis was made. The dose of ceftriaxone was increased to 2 g twice daily, and oral levofloxacin (250 mg/day) was added. Insulin therapy was initiated to manage the hyperglycemia. The echocardiographic and endoscopic examination findings were unremarkable. Thereafter, the patient’s symptoms disappeared, the serological inflammation decreased, and all the subsequent repeated BCs remained sterile. The periaortic soft tissue lesion appeared stable on serial CT imaging (Figures 2A–C). Nevertheless, serial unenhanced magnetic resonance angiography (MRA) with thin-slab maximum intensity projection (TS-MIP) demonstrated acutely progressive morphological changes of the abdominal aorta characterized by multilobular and saccular features strongly suggestive of infectious pseudoaneurysm formation (Figures 2D–F). Additionally, an aortic mural thrombus was observed on the anterior wall of the descending thoracic aorta (Supplementary Figure 1A). She was treated accordingly with anticoagulation therapy (apixaban, 2.5 mg twice daily).

**FIGURE 3:**
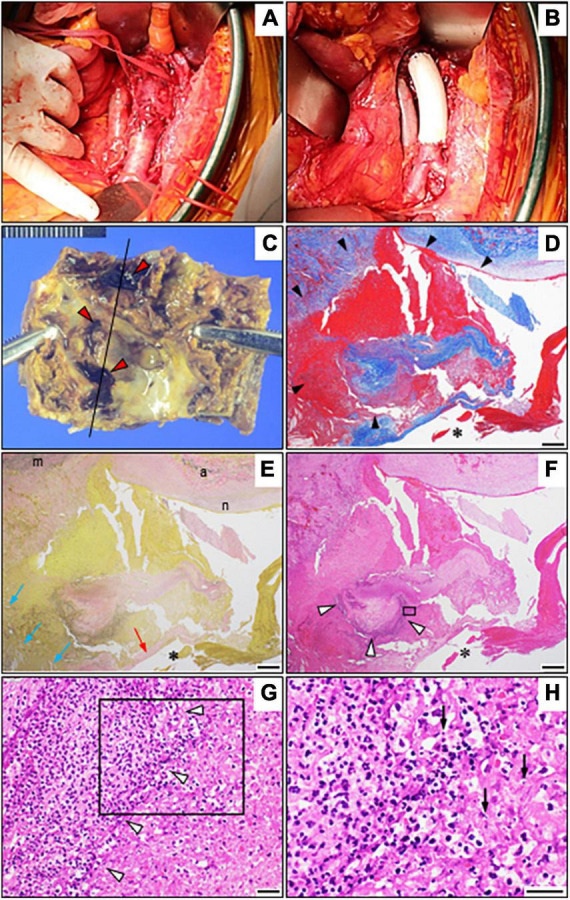
Operative view and pathological findings. **(A,B)** Intraoperative view before **(A)** and after **(B)** the repair using a Dacron graft. **(C)** The resected abdominal aortic aneurysm shows severely calcified plaque as well as several recesses with adherent mural thrombi (red arrowheads). **(D–F)** Photomicrographs of the resected abdominal aorta (longitudinal sections around the black line in **C**) with Masson’s trichrome stain **(D)**, Elastica van Gieson staining **(E)**, and hematoxylin and eosin staining **(F)** showing marked inflammatory infiltration of the aortic wall (white arrowheads) and pseudoaneurysm formation (black arrowheads) due to loss of the normal structure of the aortic wall as well as cholesterol crystals (blue arrows). **(G)** A high-power view with hematoxylin and eosin staining showing intense perivascular inflammatory infiltrates (magnified view of the black box in **F**) but no microorganisms (white arrowheads). **(H)** Notably, the magnified view of the black box in **(G)** shows the accumulation of nuclear debris (arrows). Bars: **(D–F)**, 500 μm; and **(G,H)**, 20 μm. *Vascular lumen. The red arrow depicts a fibrous cap, while “m,” “a,” and “n” depict the media, adventitia, and neointima, respectively.

Owing to the elevated risk of aortic rupture, early surgery was planned by a multidisciplinary team after confirmation of a stable inflammatory response following antimicrobial treatment. On day 30, she underwent open surgical repair of the infected aortic lesion using an *in situ* graft (GORE-TEX Vascular Graft) (Figures 3A,B). Pathology of the excised aorta revealed severely calcified atherosclerosis with inflammatory cell infiltrations predominantly consisting of neutrophils, fresh mural thrombus, and partial pseudoaneurysm formation but without obvious bacteria (Figures 3C–3H). The accumulation of nuclear debris suggestive of infection was also noted. Despite negative Gram staining and tissue culture results for the aortic wall, *Arthrobacter russicus* sequences were obtained via 16S rRNA sequencing (Supplementary Figures 2–4), leading to the potential causative microorganism. A careful interview revealed that she was a farm worker with multiple exposures to soil and frequent episodes of foreign body ingestion related to dementia. Given the unsanitary living conditions and impaired immunity prone to aortic infections including severe atherosclerosis, diabetes, and renal failure, it was highly likely that this microorganism was the causative agent of the IAA in this patient. The early postoperative MRA revealed abnormal intensity around the vascular graft highly suggestive of vascular graft infection (Supplementary Figure 1B). Therefore, the antimicrobial therapy was switched to intravenous meropenem (0.5 g twice daily) plus vancomycin (0.5 g daily) for 60 days.

After follow-up blood examination and MRA confirmed the resolution of an inflammatory response to the infection (Supplementary Figures 1C,D), the antimicrobial therapy was de-escalated to oral cefalexin (250 mg every 8 h). Her subsequent clinical course was excellent, with no infection recurrence; however, the aortic mural thrombus size remained unchanged. On day 180, she was transferred to a nursing home for rehabilitation because of dementia and advanced disuse atrophy from prolonged bed rest. A summarized illustration of the case presentation is provided in Supplementary Figure 5.

## Discussion

Here we describe the first case of IAA caused by *A. russicus*, successfully treated using an early surgical intervention concurrent with long-term antimicrobial therapy. Our case report offers three clinical implications. First, unenhanced MRI/MRA was feasible for diagnosing IAA in our patient with renal dysfunction. An IAA refers to an arterial dilation secondary to destruction of the infected aortic wall. IAA (thoracic and abdominal) accounts for 0.7–4.5% of all aortic aneurysms (1). Most IAA cases affect elderly men over 65 years of age with severe atherosclerosis (4). Although the aortic intima is extremely resistant to infection due to its barrier mechanism, any situation that damages it is a crucial predisposing factor for aortic infection (2). Aortic infection can occur via several mechanisms: (1) bacteremic seeding of an existing intimal injury, atherosclerotic plaque, and preexisting aneurysm (5–7); (2) septic embolization of the aortic vasa vasorum (8); (3) an infected foci extending to the aortic wall (9); and (4) direct bacterial inoculation during aortic injury (10). Although the microbiological spectrum involved in IAA has evolved over time and depends on geographical conditions, *Staphylococcus* spp. (50–60%) and *Salmonella* spp. (30–40%) remain the most common causative species underlying IAA owing to their high affinity for the arterial wall (1, 11, 12).

Untreated IAA can cause rupture, aortofistulous communication, mural thrombosis, distal ischemia, sepsis, or multiorgan failure. Of these, rupture, the most fatal sequela, occurs in 50–80% of all cases, with a significantly higher mortality rate of 42% in cases of IAA with free or contained rupture vs. without rupture (1, 13). Therefore, this disease requires a prompt diagnosis and appropriate timely treatment. However, the high suspicion of IAA remains challenging due to the non-specific signs and symptoms. CE-CT is the cornerstone for diagnosing IAA because it can provide detailed information about lesion size and location as well as the surrounding structures. The typical CT findings indicative of IAA include a saccular or multilobulated aneurysm, a periaortic soft tissue mass, periaortic fat stranding, and periaortic air or fluid accumulation.

A variety of microorganisms can cause IAA. Distinct IAA phenotypes specific to each microorganism may exist because of differences in pathogenicity and virulence. Although most pathogen-specific features of IAA imaging on CT remain poorly understood, the IAA features on CT that may suggest the involvement of several specific pathogens have been reported in the following three categories: (1) characteristics of IAA: while most pathogens are associated with the rapid aneurysmal expansion, *Helicobacter cinaedi* is a low virulence bacterium that contributes to the promotion of atherosclerosis via macrophage-driven proinflammatory response and hence slowly progressive over months to years (14, 15); (2) periaortic involvement: *Clostridium septicum* can survive and proliferate in tissues under anaerobic condition that may be strongly associated with the presence of periaortic gas on CT (16). Specific feature of *Mycobacterium tuberculosis* includes enlargement of periaortic lymph nodes with central hypodensity suggesting of necrotic lymphadenopathy due to the nature of direct infection from the adjacent lymph nodes (17); and (3) surrounding organ involvement: psoas abscess and lumbar osteomyelitis are reportedly frequently associated with the IAA associated with *Salmonella* spp. (11 and 19%, respectively) (18, 19). However, we faced two diagnostic challenges to reaching an accurate diagnosis in the present case. As a result, the diagnosis was delayed.

The first diagnostic challenge was the presence of significant renal dysfunction in our patient, which required us to preclude iodinated contrast media exposure. The other diagnostic challenge was that CT findings observed in our case were similar between IAA and IgG4-RD. IgG4-RD is a life-threatening inflammatory vasculopathy that frequently affects individuals of middle to advanced ages by involving large and medium-sized vessels simultaneously, which often occurs in the infrarenal segment of the abdominal aorta (20, 21). Various serious aortic complications such as intra-abdominal bleeding, intra-aneurysmal thrombus, and abdominal aortic stenosis resulting from aneurysmal development or rupture can occur. These conditions require prompt and comprehensive treatment consisting of corticosteroid and immunosuppressant therapy, vascular graft, or surgical resection (22). Although the two distinct disorders share similar age at onset and radiological findings, it is critical to differentiate between the two because they differ significantly in terms of requisite treatment approaches. Admittedly, the unenhanced MRI/MRA scan was valuable for addressing the above issues in this case. MRI can enable soft tissue characterization and provide variable information on disease activity before aortic luminal changes occur. DWI, which can measure the random motion of water protons, is frequently used to detect acute inflammation such as acute stroke, tumors, and abscesses (23). It can also identify the active phase of inflammatory aortitis or acute infection (24, 25). Because of the limited spatial resolution of DWI, its use in combination with other MRI sequences may be valuable for evaluating aortitis without involving radiation exposure or contrast agents. In addition, unenhanced MRA can provide valuable morphological information about the aortic lumen (e.g., vessel stenosis, vessel dilation, and mural thrombus) comparable to CT angiography results (26). Because of its superior soft tissue imaging characteristics, MRA can help discriminate aortitis mimics such as aortic dissection, penetrating ulcers, or mural hematomas. Furthermore, TS-MIP of the original MRA can help clinicians screen for the development of aneurysms and monitor existing aneurysms. Indeed, the TS-MIP findings of multilobular or eccentric acute aortic expansion observed in this case were characteristic of IAA and helpful in distinguishing it from IgG4-RD. IAA and IgG4-RD can also be differentiated based on histopathological findings (22).

Moreover, ^18^F-flourodeoxyglucose positron emission tomography/computed tomography (PET/CT) is a potential alternative for evaluating IAA. Although PET/CT is very sensitive for detecting metabolic activity, its diagnostic accuracy is limited due to difficulty distinguishing IAA from inflammatory aortic aneurysm and false positive results including malignancy and non-specific findings. However, a recent retrospective study analyzing 29 patients with abdominal aneurysms who underwent both PET/CT and CE-CT reported that a combination of specific imaging features (e.g., dorsal sparing of metabolic activity or wall thickening of aneurysm in PET/CT; fat stranding or fluid collections in CE-CT) and metabolic activity (intensity and site) can identify the etiology of each aneurysm with high accuracy among abdominal aortic aneurysms (infectious, inflammatory, and non-infectious/non-inflammatory) (27, 28). Therefore, the combined use of PET/CT and CE-CT may improve the diagnostic accuracy of the IAA and further studies are warranted.

In our case, IAA was confirmed based on histologic findings and the identification of *A. russicus* 16S rRNA on the resected aortic wall. Advanced age, poorly controlled diabetes, and renal dysfunction, which are closely associated with atherosclerosis, might have provided a rich nidus for aortic infection. Therefore, this case report emphasizes the importance of considering IAA as a possible source of unexplained abdominal pain and fever among elderly patients with severe atherosclerosis and the use of unenhanced MRI/MRA to evaluate and monitor aortic diseases in patients with renal dysfunction wherein iodinated contrast media exposure is undesirable.

As a second clinical implication, to the best of our knowledge, this is the first case of IAA caused by *A. russicus*. The 16S rRNA sequencing was also helpful to identify this microorganism. *Arthrobacter* spp. (Gram-positive, aerobic, non-motile, and coryneform rods) are widely distributed in the environment, especially the soil (29). The microorganisms belong to the genus *Arthrobacter* of the family *Micrococcaceae* of the order *Micrococcales.* These bacteria are isolated from animals and wastewater materials (30). *A. russicus*, firstly reported in 2004 (31), was recovered from air in the Russian space station. The first identification of *Arthrobacter* from human specimens (urine, skin, blood, vagina, and eye) was reported in 1996 (29). The incidence of *Arthrobacter* human infections is underestimated since its biochemical identification method is based on a single strain and the bacteria are difficult to isolate (29, 32, 33).

Table 1 summarizes the *Arthrobacter*-associated cardiovascular disease cases including the present case. Two cases of *A. woluwensis* endocarditis have been reported (32, 33): although conventional phenotyping using morphological and biochemical features initially led to misidentification of the causative organism in both cases, 16S rRNA sequencing on tissue or blood culture samples ultimately succeeded in identifying *A. woluwensis* as the true causative organism of IE in both cases. Therefore, 16S rRNA sequencing was required to identify *Arthrobacter* spp. as per our case. While drug injection appeared to be the entry site in two cases of *A. woluwensis* endocarditis (Table 1), the entry site remained unclear in the present case because this seems very rare among Japanese elderly individuals. Presumably, this patient might have repeatedly developed silent *Arthrobacter* bacteremia via translocation of this microorganism from the gastrointestinal tract to the portal bloodstream after the ingesting of soil or wastewater before hospitalization.

**TABLE 1:** Literature review of *Arthrobacter*-associated cardiovascular diseases.

Author	Bernasconi E (ref no. 32)	Durand C (ref no. 33)	Present case
Report year	2004	2021	This report
Age, years, sex	39, Male	52, unknown	85, female
Underlying condition	Injectable drug use	Injectable drug use	Diabetes mellitus, renal dysfunction
Clinical symptoms	Fever, fatigue, myalgia, weight loss	Fever, weakness, weight loss	Fever, abdominal pain
Diagnosis	Endocarditis of native mitral valve	Endocarditis of mitral and aortic valves	Infectious abdominal aortic aneurysm and aortic mural thrombus of the descending thoracic aorta
Surgical intervention(s)	None	Mitral valve replacement, aortic vegetectomy	*In situ* graft replacement
Species identified	*Arthrobacter woluwensis*	*Arthrobacter woluwensis*	*Arthrobacter russicus*
Identification assay	16S rRNA sequencing analysis of blood specimen	16S rRNA sequencing analysis of tissue specimen	16S rRNA sequencing analysis of tissue specimen
Antimicrobial treatment	TEIC	OX + GM, DAP + ST, TEIC + ST, followed by LZD + ST	CTRX + LVFX, MEPM + VCM followed by CFX
Treatment outcome	Cured	Cured	Cured (despite perioperative vascular graft infection)

CFX, cefalexin; CTRX, ceftriaxone; DAP, daptomycin; GM, gentamicin; LVFX, levofloxacin; LZD, linezolid; MEPM, meropenem; OX, oxacillin; rRNA, ribosomal RNA; ST, sulfamethoxazole/trimethoprim; TEIC, teicoplanin; VCM, vancomycin.

We misdiagnosed this case as IAA caused by *K. pneumoniae* because of the initial positive urine culture and BCs for *K. pneumoniae*. However, *K. pneumoniae* is rarely the causative microorganism of IAA, with limited case reports of presumably hypervirulent strains (34, 35). Based on this patient’s clinical course, all negative blood culture results after the initial antimicrobial treatment, and the negative Gram staining and molecular examination results, we concluded that this microorganism caused transient bacteremia in our case because data were lacking to support *K. pneumoniae* as the primary causative microorganism of the IAA. Therefore, our experience with this case suggests that the microorganism yielded from initial positive BCs is not always the true causative agent of the IAA. Furthermore, the frequency of positive BCs in patients with IAA varies significantly (2, 4, 11, 19, 36–38). Given the high frequency of negative BCs (up to 50% of cases), negative BCs alone are inadequate for ruling out the presence of IAA. Additionally, Gram staining and tissue cultures of the excised specimen can be negative in patients who previously received broad-spectrum antimicrobial therapy.

Similar to the present case, molecular diagnosis by 16S rRNA sequencing may be helpful for estimating the causative microorganism in cases, where IAA is strongly suspected despite negative tissue culture and repeated BCs. Recently, metagenomic next-generation sequencing and analysis may be a promising alternative (39).

A final clinical implication is that the long-term systemic antimicrobial therapy effectively controlled postoperative vascular graft infection in this case. In principle, surgery is required to treat IAA. Surgical procedures include open surgery to remove the infected tissue with/without *in situ* graft repair, extra-anatomic bypass, and endovascular stent graft insertion. Each procedure is determined based on the infected aneurysm location, infection degree, and patient condition. Since the perioperative mortality rate for the IAA in the acute setting remains high (3), endovascular stent graft insertion can be an acceptable alternative to conventional open surgery or a bridge to subsequent open surgical repair in high-risk surgical patients with severe sepsis or hemodynamic instability. Recent nationwide studies revealed that the results of the endovascular approach were comparable to those of open surgery (40, 41). In this case, despite IAA removal with *in situ* graft repair under adequate infection control with antimicrobial agents, the patient developed a serious postoperative vascular graft infection that was fortunately controlled with long-term systemic antimicrobial therapy. However, *in situ* graft infections caused by inadequate infection clearance can lead to a poor prognosis and a high mortality rate (nearly 100%) within 2 years (42). Although a postoperative antimicrobial duration of 6 weeks to 6 months is generally recommended (1), there are no specific criteria for discontinuing antimicrobial therapy in patients with IAA. Furthermore, this patient had residual aortic mural thrombus despite receiving anticoagulation therapy. Therefore, our patient remains under long-term surveillance for recurrent infections and the risk of aortic mural thrombus–related complications.

## Limitations

Limited to small numbers, the antimicrobial susceptibility patterns among clinical isolates related to *Arthrobacter* spp. have been reported (29). However, the antimicrobial susceptibility of *Arthrobacter* was unknown in our case since we could not recover the microorganism from the tissue and blood. Therefore, the present case received empiric antimicrobial therapy. Fortunately, long-term challenging therapy (ceftriaxone and levofloxacin, meropenem and vancomycin, followed by cefalexin) resolved the clinical symptoms, imaging findings, and blood laboratory data of inflammatory responses. Choosing optimal antimicrobial therapies for *Arthrobacter* infections remains challenging because of the difficulty isolating and identifying the phenotype (32, 33).

## Future directions

Considering the difficulty of identification of *Arthrobacter* spp. by conventional biochemical approaches, the frequency of these microorganism infections in patients with IAA may remain underestimated. Therefore, prospective evaluation of the complementary use of molecular testing for the identification of *Arthrobacter* infection in all cases of IAA is further warranted.

## Conclusion

Herein, we report the first case of IAA caused by *A. russicus* that was successfully treated with a combination of surgery and long-term antimicrobial therapy. Unenhanced MRA/MRI was useful for identifying IAA and determining its treatment response in this patient with renal dysfunction. In addition, 16S rRNA sequencing analysis allowed us to identify *A. russicus* as the causative organism of IAA. *A. russicus* can cause IAA, but is difficult to identify by conventional biochemical characterization. Therefore, a molecular diagnosis should be performed to identify the causative microorganism in patients with culture-negative IAA, considering the possibility of *A. russicus.*

## Data availability statement

The datasets presented in this study can be found in online repositories. The names of the repository and accession number(s) can be found below: NCBI GenBank; LC715707 and LC715708.

## Author contributions

HY contributed to the clinical design and study concept. HY, TS, TH, and HO acquired the clinical data. YF and MG performed the microbiological analyses. YI performed the pathological analyses. JI performed the radiological analyses. HY, JI, and TT interpreted the data and drafted the manuscript. All authors discussed, read, and approved the manuscript and its submission for publication.

## Abbreviations

BCs: blood cultures
CE-CT: contrast-enhanced computed tomography
CT: computed tomography
DWI: diffusion-weighted imaging
Ga-SPECT: gallium-67 single-photon emission computed tomography
IgG4-RD: immunoglobulin G4-related disease
IAA: infectious aortic aneurysm
MRA: magnetic resonance angiography
MRI: magnetic resonance imaging
PET/CT: ^18^F-flourodeoxyglucose positron emission tomography/computed tomography
rRNA: ribosomal RNA
TS-MIP: thin-slab maximum intensity projection.

## References

[1] Wilson WR Bower TC Creager MA Amin-Hanjani S O’Gara PT Lockhart PB. Vascular graft infections, mycotic aneurysms, and endovascular infections: a scientific statement from the American Heart Association. *Circulation.* (2016) 134:e412–60. 10.1161/CIR.0000000000000457 27737955

[2] Oderich GS Panneton JM Bower TC Cherry KJ Jr. Rowland CM Noel AA. Infected aortic aneurysms: aggressive presentation, complicated early outcome, but durable results. *J Vasc Surg.* (2001) 34:900–8. 10.1067/mva.2001.118084 11700493

[3] Kim HH Kim DJ Joo HC. Outcomes of open repair of mycotic aortic aneurysms with in situ replacement. *Korean J Thorac Cardiovasc Surg.* (2017) 50:430–5. 10.5090/kjtcs.2017.50.6.430 29234609 PMC5716645

[4] Lopes RJ Almeida J Dias PJ Pinho P Maciel MJ. Infectious thoracic aortitis: a literature review. *Clin Cardiol.* (2009) 32:488–90. 10.1002/clc.20578 19743492 PMC6653599

[5] Reddy DJ Shepard AD Evans JR Wright DJ Smith RF Ernst CB. Management of infected aortoiliac aneurysms. *Arch Surg.* (1991) 126:873–8. 1854247 10.1001/archsurg.1991.01410310083012

[6] Itatani K Miyata T Komiyama T Shigematsu K Nagawa H. An ex-situ arterial reconstruction for the treatment of an infected suprarenal abdominal aortic aneurysm involving visceral vessels. *Ann Vasc Surg.* (2007) 21:380–3. 10.1016/j.avsg.2006.06.013 17484976

[7] Ernst CB Campbell HC Jr. Daugherty ME Sachatello CR Griffen WO Jr. Incidence and significance of intra-operative bacterial cultures during abdominal aortic aneurysmectomy. *Ann Surg.* (1977) 185:626–33. 10.1097/00000658-197706000-00003 324416 PMC1396230

[8] Shaikholeslami R Tomlinson CW Teoh KH Molot MJ Duke RJ. Mycotic aneurysm complicating staphylococcal endocarditis. *Can J Cardiol.* (1999) 15:217–22. 10079782

[9] Hsu RB Lin FY. Psoas abscess in patients with an infected aortic aneurysm. *J Vasc Surg.* (2007) 46:230–5. 10.1016/j.jvs.2007.04.017 17600660

[10] Samore MH Wessolossky MA Lewis SM Shubrooks SJ Jr. Karchmer AW. Frequency, risk factors, and outcome for bacteremia after percutaneous transluminal coronary angioplasty. *Am J Cardiol.* (1997) 79:873–7. 10.1016/s0002-9149(97)00006-4 9104897

[11] Brown SL Busuttil RW Baker JD Machleder HI Moore WS Barker WF. Bacteriologic and surgical determinants of survival in patients with mycotic aneurysms. *J Vasc Surg.* (1984) 1:541–7. 6436514

[12] Hsu RB Tsay YG Wang SS Chu SH. Surgical treatment for primary infected aneurysm of the descending thoracic aorta, abdominal aorta, and iliac arteries. *J Vasc Surg.* (2002) 36:746–50. 10.1067/mva.2002.126557 12368720

[13] Müller BT Wegener OR Grabitz K Pillny M Thomas L Sandmann W. Mycotic aneurysms of the thoracic and abdominal aorta and iliac arteries: experience with anatomic and extra-anatomic repair in 33 cases. *J Vasc Surg.* (2001) 33:106–13. 10.1067/mva.2001.110356 11137930

[14] Khan S Rahman HN Okamoto T Matsunaga T Fujiwara Y Sawa T. Promotion of atherosclerosis by *Helicobacter* cinaedi infection that involves macrophage-driven proinflammatory responses. *Sci Rep.* (2014) 15:4680. 10.1038/srep04680 24732347 PMC3986732

[15] Kushimoto K Yonekura R Umesue M Oshiro Y Yamasaki H Yoshida K. Infected thoracic aortic aneurysm caused by *Helicobacter cinaedi*. *Ann Vasc Dis.* (2017) 10:139–42. 10.3400/avd.cr.16-00126 29034040 PMC5579772

[16] Ito F Inokuchi R Matsumoto A Kumada Y Yokoyama H Ishida T. Presence of periaortic gas in Clostridium septicum-infected aortic aneurysm aids in early diagnosis: a case report and systematic review of the literature. *J Med Case Rep.* (2017) 11:268. 10.1186/s13256-017-1422-0 28931420 PMC5607595

[17] Mearelli F Burekovic I Zanetti M Altamura N Carlo G Biolo G. Disseminated tuberculosis in an immunocompetent patient. *Int J Infect Dis.* (2013) 17:e784–6. 10.1016/j.ijid.2013.02.026 23931745

[18] Guo Y Bai Y Yang C Wang P Gu L. Mycotic aneurysm due to *Salmonella* species: clinical experiences and review of the literature. *Braz J Med Biol Res.* (2018) 51:e6864. 10.1590/1414-431X20186864 29947649 PMC6040868

[19] Soravia-Dunand VA Loo VG Salit IE. Aortitis due to *Salmonella*: report of 10 cases and comprehensive review of the literature. *Clin Infect Dis.* (1999) 29:862–8. 10.1086/520450 10589904

[20] Ozawa M Fujinaga Y Asano J Nakamura A Watanabe T Ito T. Clinical features of IgG4-related periaortitis/periarteritis based on the analysis of 179 patients with IgG4-related disease: a case-control study. *Arthritis Res Ther.* (2017) 19:223. 10.1186/s13075-017-1432-8 28978347 PMC5628426

[21] Perugino CA Wallace ZS Meyersohn N Oliveira G Stone JR Stone JH. Large vessel involvement by IgG4-related disease. *Medicine.* (2016) 95:e3344. 10.1097/MD.0000000000003344 27428181 PMC4956774

[22] Yamamoto H Ito Y Isogai J Ishibashi-Ueda H Nakamura Y. Immunoglobulin G4-related multiple giant coronary artery aneurysms and a single left gastric artery aneurysm. *JACC Case Rep.* (2020) 2:769–74. 10.1016/j.jaccas.2020.03.015 34317345 PMC8301683

[23] Andre JB Bammer R. Advanced diffusion-weighted magnetic resonance imaging techniques of the human spinal cord. *Top Magn Reson Imaging.* (2010) 21:367–78. 10.1097/RMR.0b013e31823e65a1 22158130 PMC3985837

[24] Doi S Kuroiwa Y Kusumoto K Yamashita A Furukoji E Tai H. Therapeutic response of immunoglobulin 4-related aortitis and pancreatitis demonstrated by diffusion-weighted MRI. *Radiol Case Rep.* (2019) 14:1132–5. 10.1016/j.radcr.2019.06.020 31360274 PMC6637269

[25] Dumont RA Keen NN Bloomer CW Schwartz BS Talbott J Clark AJ. Clinical utility of diffusion-weighted imaging in spinal infections. *Clin Neuroradiol.* (2019) 29:515–22. 10.1007/s00062-018-0681-5 29582111 PMC6158113

[26] Hartlage GR Palios J Barron BJ Stillman AE Bossone E Clements SD. Multimodality imaging of aortitis. *JACC Cardiovasc Imaging.* (2014) 7:605–19. 10.1016/j.jcmg.2014.04.002 24925329

[27] Husmann L Huellner MW Ledergerber B Eberhard N Kaelin MB Anagnostopoulos A. Diagnostic accuracy of PET/CT and contrast enhanced CT in patients with suspected infected aortic aneurysms. *Eur J Vasc Endovasc Surg.* (2020) 59:972–81. 10.1016/j.ejvs.2020.01.032 32340877

[28] Husmann L Huellner MW Gruenig H Ledergerber B Messerli M Mestres CA. Imaging characteristics and diagnostic accuracy of FDG-PET/CT, contrast enhanced CT and combined imaging in patients with suspected mycotic or inflammatory abdominal aortic aneurysms. *PLoS One.* (2022) 17:e0272772. 10.1371/journal.pone.0272772 35944039 PMC9362916

[29] Funke G Hutson RA Bernard KA Pfyffer GE Wauters G Collins MD. Isolation of *Arthrobacter* spp. from clinical specimens and description of *Arthrobacter cumminsii* sp. nov. and *Arthrobacter woluwensis* sp. nov. *J Clin Microbiol.* (1996) 34:2356–63. 10.1128/jcm.34.10.2356-2363.1996 8880479 PMC229268

[30] Funke G von Graevenitz A Clarridge JE III Bernard KA. Clinical microbiology of coryneform bacteria. *Clin Microbiol Rev.* (1997) 10:125–59. 10.1128/CMR.10.1.125 8993861 PMC172946

[31] Li Y Kawamura Y Fujiwara N Naka T Liu H Huang X. *Rothia aeria* sp. nov., *Rhodococcus baikonurensis* sp. nov. and *Arthrobacter russicus* sp. nov., isolated from air in the Russian space laboratory Mir. *Int J Syst Evol Microbiol.* (2004) 54:827–35. 10.1099/ijs.0.02828-0 15143031

[32] Bernasconi E Valsangiacomo C Peduzzi R Carota A Moccetti T Funke G. *Arthrobacter woluwensis* subacute infective endocarditis: case report and review of the literature. *Clin Infect Dis.* (2004) 38:e27–31. 10.1086/381436 14765360

[33] Durand C Kouchit Y Prots L Degand N Dellamonica P Demonchy E. A case of infective endocarditis caused by *Arthrobacter woluwensis*. *Eur J Clin Microbiol Infect Dis.* (2021) 40:1329–31. 10.1007/s10096-021-04154-0 33432493

[34] Chuang YC Lee MF Yu WL. Mycotic aneurysm caused by hypermucoviscous *Klebsiella pneumoniae* serotype K54 with sequence type 29: an emerging threat. *Infection.* (2013) 41:1041–4. 10.1007/s15010-013-0447-6 23508461

[35] Chen YJ Chen SY Wang JT Hsueh PR. Mycotic aneurysm caused by gas-forming serotype K5 *Klebsiella pneumoniae*. *Int J Infect Dis.* (2009) 13:e47–8. 10.1016/j.ijid.2008.06.008 18774741

[36] Sörelius K Budtz-Lilly J Mani K Wanhainen A. Systematic review of the management of mycotic aortic aneurysms. *Eur J Vasc Endovasc Surg.* (2019) 58:426–35. 10.1016/j.ejvs.2019.05.004 31320247

[37] Hsu RB Lin FY. Surgical pathology of infected aortic aneurysm and its clinical correlation. *Ann Vasc Surg.* (2007) 21:742–8. 10.1016/j.avsg.2007.01.015 17499963

[38] Bennett DE. Primary mycotic aneurysms of the aorta. Report of case and review of the literature. *Arch Surg.* (1967) 94:758–65. 10.1001/archsurg.1967.01330120012004 6071707

[39] Huang ZD Zhang ZJ Yang B Li WB Zhang CJ Fang XY. Pathogenic detection by metagenomic next-generation sequencing in osteoarticular infections. *Front Cell Infect Microbiol.* (2020) 10:471. 10.3389/fcimb.2020.00471 33042860 PMC7527540

[40] Sörelius K Mani K Björck M Sedivy P Wahlgren CM Taylor P. Endovascular treatment of mycotic aortic aneurysms: a European multicenter study. *Circulation.* (2014) 130:2136–42. 10.1161/CIRCULATIONAHA.114.009481 25378548

[41] Sörelius K Wanhainen A Furebring M Björck M Gillgren P Mani K. Nationwide study of the treatment of mycotic abdominal aortic aneurysms comparing open and endovascular repair. *Circulation.* (2016) 134:1822–32. 10.1161/CIRCULATIONAHA.116.024021 27799273

[42] Batt M Jean-Baptiste E O’Connor S Bouillanne PJ Haudebourg P Hassen-Khodja R. In-situ revascularisation for patients with aortic graft infection: a single centre experience with silver coated polyester grafts. *Eur J Vasc Endovasc Surg.* (2008) 36:182–8. 10.1016/j.ejvs.2008.02.013 18440252

