# Supplementary material for: Decisive diagnostic clue for infectious abdominal aortic aneurysm caused by Arthrobacter russicus in a diabetic elderly woman with renal dysfunction: A case report and literature review
Source: Front Cardiovasc Med. 2022 Oct 28;9:1007213. doi: 10.3389/fcvm.2022.1007213 (PMC9650533; doi:10.3389/fcvm.2022.1007213)
Supplement: Supplementary file 1 [file Data_Sheet_1.DOCX]

Supplementary Material

**TEXT LEGENDS**

**Text 1. Microbiological analyses**

A sample tube (containing the affected abdominal aortic tissue resected in saline buffer) was immediately sent to the Laboratory of Infectious Diseases, Graduate School of Infection Control Sciences & Ōmura Satoshi Memorial Institute, Kitasato University. The cap of the sample tube was tightly closed using parafilm wrapping (**Supplementary Figure 2A, arrows**). This tube contained three samples of incised aortic tissue, pellets, and suspensions (**Supplementary Figure 2, AO1, AO2, and AO3, respectively)**. We conducted the incision procedure in our safety cabinet to minimize tissue contamination (**Supplementary Figure 2B)**. The incision line is shown in white color. We extracted three samples, each from the incised aorta, pellet, and suspension, using the DNeasy Blood and Tissue Kit (Qiagen, Germany) according to the manufacturer’s protocol, as previously described (1). We measured DNA concentrations, total amounts, and quality (absorbance 260/280 ratio) (**Supplementary Figure 2C)**, and the three DNA samples from tissue, pellet, and suspension were assigned to AO1, AO2, and AO3, respectively. To obtain viable causative agent(s), we performed Gram staining imaging (**Supplementary Figure 3A)** and isolation culture using soft agar media (including Todd–Hewitt broth supplemented with yeast extract and brain heart infusion) (**Supplementary Figure 3B)** using the pellet. The multi-nucleated leukocytes (arrows) in the absence of bacteria were noted. The culture yielded no bacterial growth 4 weeks after the start of inoculation.

For molecular diagnosis, we performed polymerase chain reaction (PCR)-based amplification of bacterial 16S rRNA using the following universal primer set with Ex-Taq Hot Start DNA Polymerase (TaKaRa, Japan): 27F (5'–AGAGTTTGATCMTGGCTCAG–3')/1485R (5'–TACGGTTACCTTGTTACGAC–3'), as described previously (2–4). The expected amplicons (approximately 1,500-bp) were found by electrophoresis of PCR products on 1.5% agarose gel (**Supplementary Figure 3C)**. Purified amplicons were obtained using the Qiaquick PCR Purification Kit (Qiagen), according to the manufacturer’s protocol. The concentration and quality of the purified amplicons (260/280 ratio) were measured (**Supplementary Figure 3D**). The same primers (27F/1485R) were used for direct sequencing using the DNA template (AO1) from aortic tissue, and sequencing was performed to identify the causative bacterial species. DNA samples were unambiguously identified based on only one choice with ≥98.7% similarity to the 16S rRNA sequence of the bacterial type strain using the nucleotide-nucleotide Basic Local Alignment Search Tool (https://blast.ncbi.nlm.nih.gov/Blast.cgi) available on the National Center for Biotechnology Information website (2–4). The query results show our original nucleotide sequence (655-bp) using 27F sequencing primer: the subject shows a partial 16S rRNA gene sequence of *Arthrobacter russicus* type strain DSM 14555 (1,508-bp, GenBank Accession No. MG946228.1) (5). We found high percent similarity (99.2%, 650-bp/655-bp, GenBank Accession No. LC715707 through the DNA Data Bank of Japan [DDBJ], Entry ID 62a68de53a01a5005faa9d85.soshiki27F) (**Supplementary Figure 4)**. Additionally, the query results show our original nucleotide sequence (657-bp) using 1485R sequencing primer: the subject shows partial 16S rRNA gene sequence of *Arthrobacter russicus* type strain DSM 14555 (1,508-bp, GenBank Accession No. MG946228.1) (5). A high percentage similarity (99.7%, 655-bp/657-bp, GenBank Accession No. LC715708 through DDBJ entry ID 62a68de53a01a5005faa9d85. soshiki1485R) was noted. Moreover, direct sequencing by 1485R was performed using a DNA template (AO3) from the suspension. The nucleotide sequence was identical to the sequencing results obtained using the AO1 DNA template. We conclude that *Arthrobacter* *russicus* can be genetically identified based on 16S rRNA sequencing data using DNA templates from the affected aortic tissue and suspension.

**References**

1. Yamamoto H, Yamada H, Maeda T, Goto M, Ikeda Y, Takahashi T. Miniature erupting volcano-shaped mitral valve aneurysm secondary to *Streptococcus agalactiae* ST1656 endocarditis: A case report. Front Cardiovasc Med. (2021) 8:728792. doi: 10.3389/fcvm.2021.728792
2. Kakuta R, Yano H, Hidaka H, Miyazaki H, Irimada M, Oda K, et al. Severe acute otitis media caused by mucoid *Streptococcus pyogenes* in a previously healthy adult. Tohoku J Exp Med. (2014) 232:301–4. doi: 10.1620/tjem.232.301
3. Fukushima Y, Tsuyuki Y, Goto M, Yoshida H, Takahashi T. Species identification of β-hemolytic streptococci from diseased companion animals and their antimicrobial resistance data in Japan (2017). Jpn J Infect Dis. (2019) 72:94–8. doi: 10.7883/yoken.JJID.2018.231
4. Kurita G, Tsuyuki Y, Shibata S, Itoh M, Goto M, Yoshida H, et al. Species identification of β-hemolytic streptococci from diseased companion animals and their antimicrobial resistance patterns in Japan (2021). Jpn J Vet Res. (2022) 70:19–28. doi: 10.14943/jjvr.70.1.19
5. Li Y, Kawamura Y, Fujiwara N, Naka T, Liu H, Huang X, et al. *Rothia aeria* sp. nov., *Rhodococcus baikonurensis* sp. nov. and *Arthrobacter russicus* sp. nov., isolated from air in the Russian space laboratory Mir. Int J Syst Evol Microbiol. (2004) 54:827–35. doi: 10.1099/ijs.0.02828-0

**SUPPLEMENTARY FIGURE LEGENDS**

**Supplementary Figure legends**

**Supplementary Figure 1. MRA** **imaging assessment of the pre- and postoperative infectious abdominal aorta.**

(**A**) On day 11 of admission, the follow-up MRA shows an aortic mural thrombus of the descending thoracic aorta (arrow). (**B–D**) Serial postoperative MRA images show a gradual improvement and resolution of the heterogeneous perigraft hyperintense signals (arrowheads) observed in the perioperative period, as shown in **Suppl. Fig. 1B**. POD, postoperative day.

**Supplementary Figure 2. Tissue preparation for molecular diagnosis.**

(**A**) Aorta, pellet, and suspension. (**B**) An incision on the intimal surface for DNA extraction. (**C**) The corresponding DNA concentrations, total amounts, and quality.

**Supplementary Figure 3. Staining and isolation, with bacterial rRNA amplification.**

(**A, B**) Gram staining image and a culture image of soft agar media using the pellet. Bar, 5 μm. (**C**) Electrophoretic image of the 16S rRNA amplicons in 1.5% agarose gel. Lanes 1–3, three amplicons from AO1–AO3 DNAs. M, size marker. (**D**) Purified amplicon concentration and quality.

**Supplementary Figure 4. Bacterial rRNA sequencing.**

The 16S rRNA sequences were amplified using a forward primer (left side) and reverse primer (right side). Query shows the original nucleotide sequence: the subject shows the 16S rRNA sequence of *Arthrobacter russicus* type strain.

**Supplementary Figure 5. Timeline of case presentation**

**Abbreviations.**

CFX, cefalexin; CTRX, ceftriaxone; CT, computed tomography; Ga-SPECT, gallium-67 single-photon emission computed tomography; IAA, infectious aortic aneurysm; IV, intravenous; MRA, magnetic resonance angiography; MRI, magnetic resonance imaging; MEPM, meropenem; LVFX, levofloxacin; rRNA, ribosomal RNA; TS-MIP, thin-slab maximum intensity projection; VCM, vancomycin.
