# Supplementary material for: Decisive diagnostic clue for infectious abdominal aortic aneurysm caused by Arthrobacter russicus in a diabetic elderly woman with renal dysfunction: A case report and literature review
Source: Front Cardiovasc Med. 2022 Oct 28;9:1007213. doi: 10.3389/fcvm.2022.1007213 (PMC9650533; doi:10.3389/fcvm.2022.1007213)
Supplement: Supplementary file 2 [file Data_Sheet_2.PDF]

CARE case report guidelines

CARE Checklist of information to include when writing a case report

(cc) BY-NC-ND

Topic	Item	Checklist item description	Reported on Line
Title	1	The diagnosis or intervention of primary focus followed by the words "case report"	1-3
Key Words	2	2 to 5 key words that identify diagnoses or interventions in this case report, including "case report"	20-21
Abstract (no references)	3a	Introduction: What is unique about this case and what does it add to the scientific literature?	32-37
	3b	Main symptoms and/or important clinical findings	37-46
	3c	The main diagnoses, therapeutic interventions, and outcomes	46-50
	3d	Conclusion—What is the main "take-away" lesson(s) from this case?	50-53
Introduction	4	One or two paragraphs summarizing why this case is unique (**may include references**)	68-72
Patient Information	5a	De-identified patient specific information.	75
	5b	Primary concerns and symptoms of the patient.	75-76
	5c	Medical, family, and psycho-social history including relevant genetic information	76
	5d	Relevant past interventions with outcomes	N/A
Clinical Findings	6	Describe significant physical examination (PE) and important clinical findings.	77-81
Timeline	7	Historical and current information from this episode of care organized as a timeline	N/A
Diagnostic Assessment	8a	Diagnostic testing (such as PE, laboratory testing, imaging, surveys).	81-86, 88-90, 94-99, 109-114
	8b	Diagnostic challenges (such as access to testing, financial, or cultural)	94-95, 102-105
	8c	Diagnosis (including other diagnoses considered)	86-87, 91-92, 100-101, 119-129
	8d	Prognosis (such as staging in oncology) where applicable	N/A
Therapeutic Intervention	9a	Types of therapeutic intervention (such as pharmacologic, surgical, preventive, self-care)	91-92, 105-106, 114-119, 131-135
	9b	Administration of therapeutic intervention (such as dosage, strength, duration)	92, 105-106, 114-115, 132, 135
	9c	Changes in therapeutic intervention (with rationale)	91-92, 102-106, 113-115, 129-135
Follow-up and Outcomes	10a	Clinician and patient-assessed outcomes (if available)	133-134
	10b	Important follow-up diagnostic and other test results	133-136
	10c	Intervention adherence and tolerability (How was this assessed?)	N/A
	10d	Adverse and unanticipated events	129-131
Discussion	11a	A scientific discussion of the strengths AND limitations associated with this case report	142-294
	11b	Discussion of the relevant medical literature **with references**.	145-292
	11c	The scientific rationale for any conclusions (including assessment of possible causes)	142-294
	11d	The primary "take-away" lessons of this case report (without references) in a one paragraph conclusion	300-303
Patient Perspective	12	The patient should share their perspective in one to two paragraphs on the treatment(s) they received	N/A
Informed Consent	13	Did the patient give informed consent? Please provide if requested	Yes ☑ No ☐
