# Supplementary figures and images for: Decisive diagnostic clue for infectious abdominal aortic aneurysm caused by Arthrobacter russicus in a diabetic elderly woman with renal dysfunction: A case report and literature review
Source: Front Cardiovasc Med. 2022 Oct 28;9:1007213. doi: 10.3389/fcvm.2022.1007213 (PMC9650533; doi:10.3389/fcvm.2022.1007213)

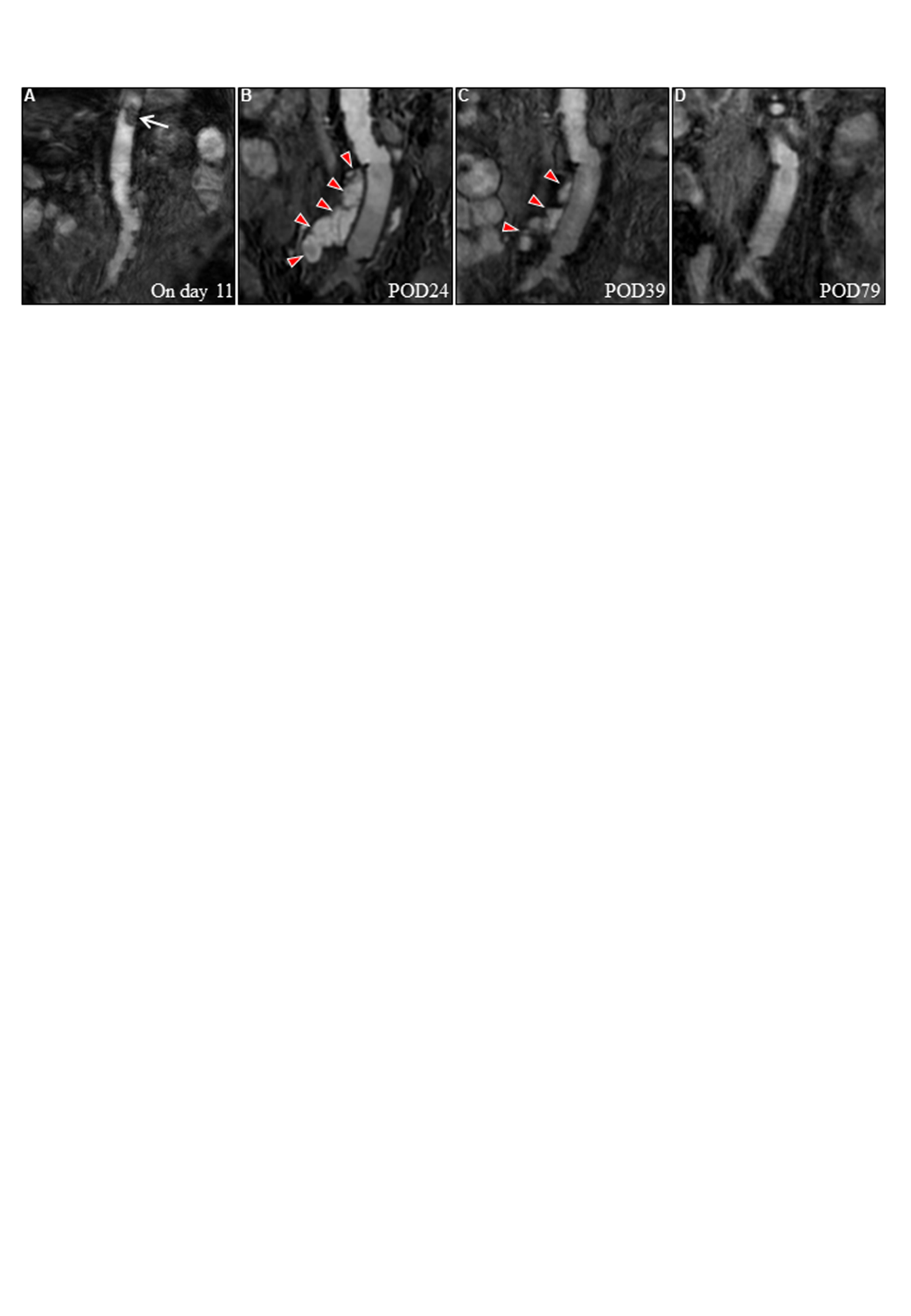

Supplement: Supplementary file 3 [file Image_1.TIF]

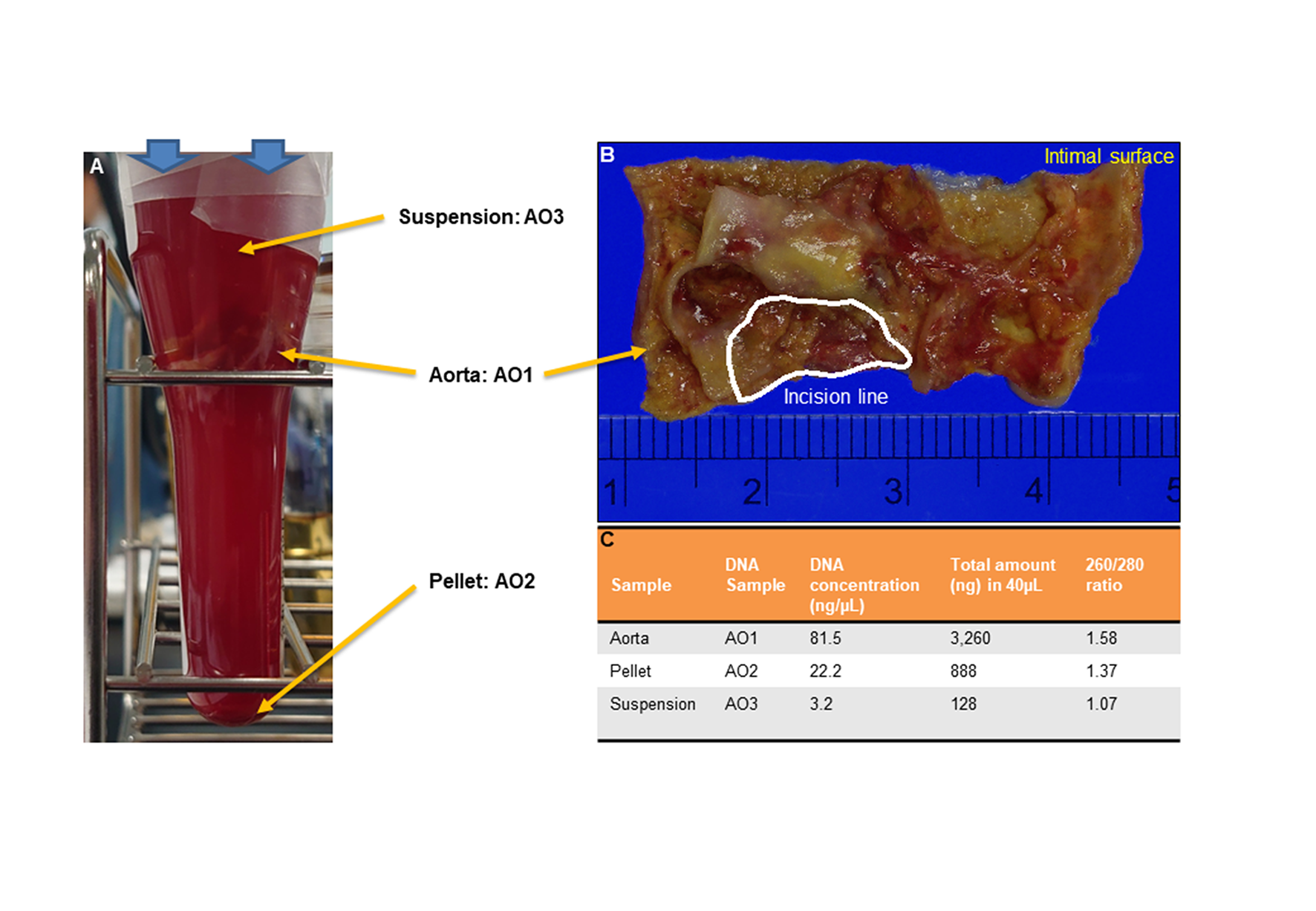

Supplement: Supplementary file 4 [file Image_2.TIF]

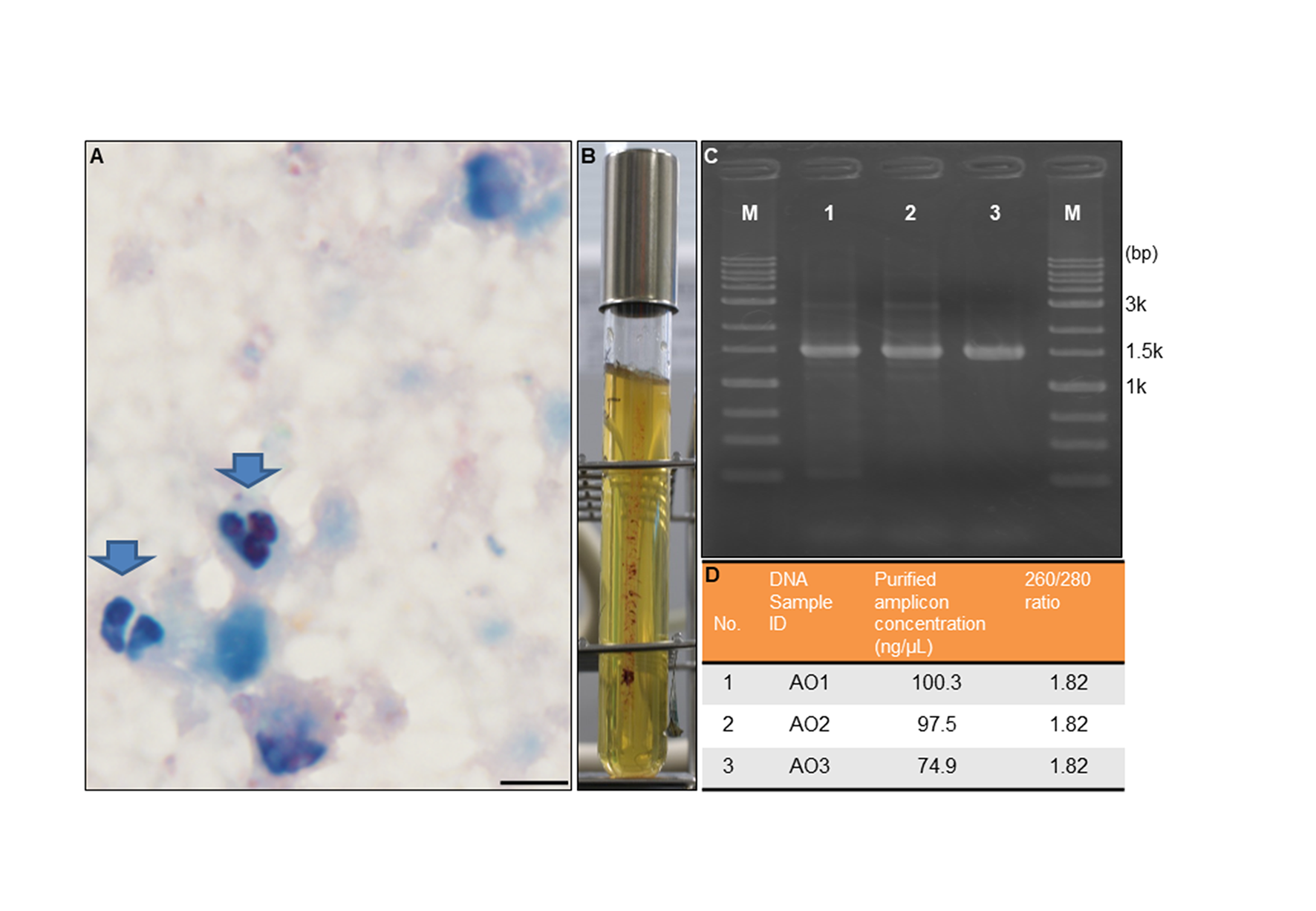

Supplement: Supplementary file 5 [file Image_3.TIF]

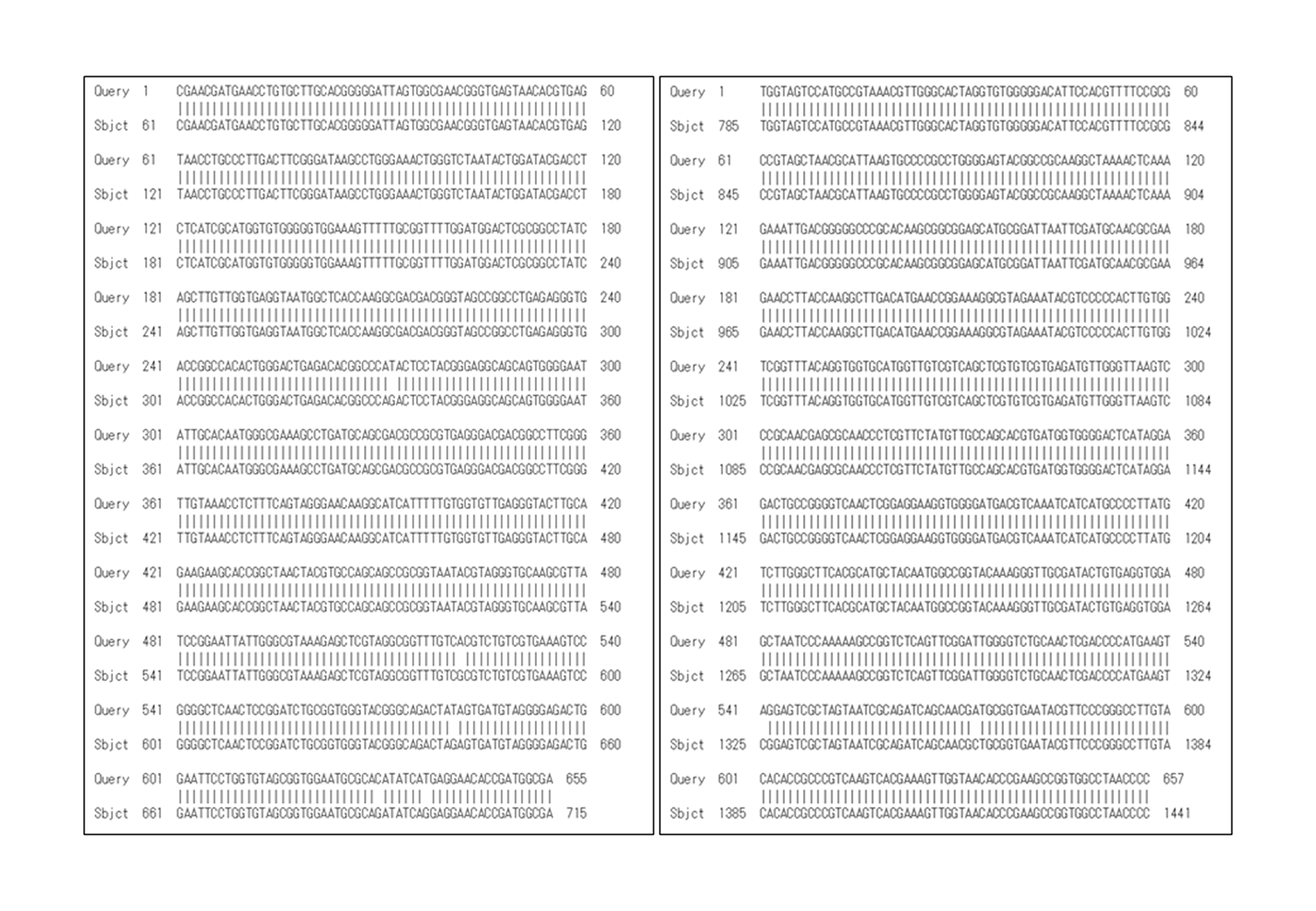

Supplement: Supplementary file 6 [file Image_4.TIF]

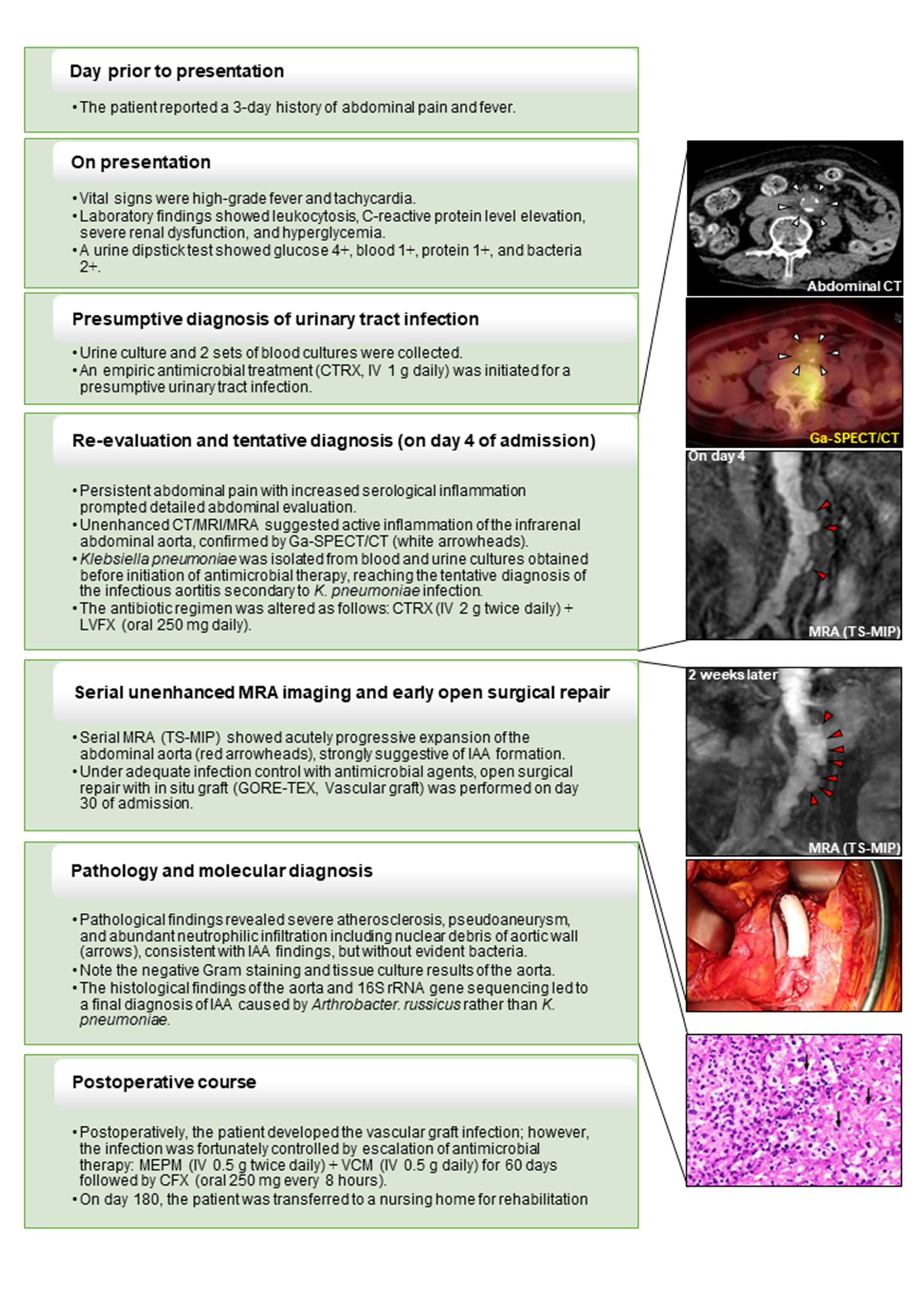

Supplement: Supplementary file 7 [file Image_5.TIF]
